# Foreign Summary

**Published:** 1839-06-29

**Authors:** 


### FOREIGN SUMMARY.

*Clinical Lecture on Pulmonary Consumption.* By ROBERT CARSWELL, M. D.—Gentlemen: I am desirous, to-day, of directing your attention to two cases of phthisis lately admitted to the male ward, both of which are highly deserving of your attention, on account of the differences which they present in regard to the probable origin of the disease, its progress, and the morbid phenomena by which its presence is recognised in each.

The first patient, James Shirley, 36 years of age, of a stout, muscular form, states that he never had a day's illness before the present attack, which was only two months and a half ago, although the disease has already arrived at the commencement of the second period; that is to say, the softening of the tuberculous matter, and the formation of tubercular excavations.

The second patient, William Calvert, aged 40, of a slight habit of body, and nervous temperament, dates the commencement of the disease to a somewhat later date; yet, in this patient, it may be said to be only at the commencement of the first stage; that is to say, the material or essential anatomical element of phthisis, the tuberculous matter, is yet in that state which is generally denominated the state of crudity. Under these circumstances, and as the physical signs of phthisis pulmonalis are derivable from the presence of the tuberculous matter, and the morbid conditions to which, as a foreign body, it gives rise in the pulmonary tissue, it is obvious that these signs must, in these two cases, be considerably modified; and so much so is this the case, that whilst in the first patient, in whom the disease is advanced and progressing rapidly, the physical signs are marked and readily detected by percussion and auscultation; in the second these are so slightly announced by these means, that an ordinary observer might overlook them altogether. The existence of phthisis in this patient is, however, not less certain than in the former,—as certain, indeed, as if we heard those sounds which indicate the presence of tubercular excavations. I shall presently state the grounds on which the certainty of our diagnosis is founded in this and similar cases. In the mean time I shall briefly state the case of William Calvert, the patient in whom we have the example of incipient phthisis.

This patient, 40 years of age, was admitted the 13th of this month. He is a tailor by profession; rather of regular habits; both of his parent died of some affection of the chest; he has brothers and sisters who are all healthy; he himself has always been delicate. At the age of 18 he became subject to colds; fourteen years ago he had rheumatic gout; when a boy he had frequent attacks of epistaxis. *Three* months ago, having caught a fresh cold from coming out of a warm workshop into the cold night air, he became subject to a cough, more severe than any preceding one, and which was accompanied by occasional hæmoptysis. The blood spit up was usually in streaks in the sputa, but sometimes in clots. The hæmoptysis, when it first appeared, continued for two days, but was not very profuse. It has gradually dimished in quantity, and became fainter in colour since the commencement of the attack.

On his admission he presented the following symptoms:—Pain and constriction across the chest; headach; considerable heat of skin; profuse perspirations at night; alternations of heat and cold; occasional rigors; weakness and pains in the limbs, especially in the knees, which are worse when he is hot. On percussion the sound is *dull* on the right ride, under the clavicle; in the same situation, on the opposite side, it is natural. On the same side, viz., the right, on which the sound is dull on percussion, the respiratory murmur is *diminished in intensity*, and is somewhat *rough* or *harsh.* This character of the respiratory sound accompanies not only *inspiration*, but also *expiration*; and, besides, the expiratory sound is greatly *increased in intensity*, being nearly equal to that which accompanies inspiration. On the left side, that on which the sound on percussion is clear, the respiratory sound is, perhaps, stronger than natural; *expiration*, however, not being accompanied by a proportionate increase in intensity of the sound to which it gives rise; the breathing is short; pulse 76; appetite bad; bowels confined; flatulency; urine plentiful, but at times high coloured.

Taking a general view of this case, we find in it a good illustration of the manner in which the phenomena of phthisis pulmonalis are successively developed, more especially where there is an hereditary predisposition to the disease, as was probably the case in our patient, whose parents are said to have died of a chest affection, and notwithstanding that his brothers and sisters are stated to be in good health. From his boyhood upwards he has been delicate and subject to colds, and has not got rid of the cough with which he became affected three months ago. This last cough has been accompanied by hæmoptysis, the blood appeared in the sputa, either in streaks or clots. This has gradually diminished, and ultimately disappeared; but it has been succeeded by various constitutional derangements, chiefly febrile symptoms of a hectic character, viz., alternation of heat and cold, occasional rigors, and profuse perspirations. Of these precursory symptoms by far the greatest amount of value, in a diagnostic point of view, is to be attached to the hæmoptysis, as predicating the existence of tubercular phthisis. Its occurrence in this case along with, and after repeated attacks of cough, increases its diagnostic value; and taken in connection with the local signs which I have enumerated as existing in the right side of the chest, we cannot hesitate to attribute its occurrence to the presence of tubercles in the lungs; as indicating, in fact, three months ago, the existence of tubercular phthisis in this patient. In attaching so much importance to hæmoptysis, I do not mean to convey the idea that it may not frequently occur, and to a great extent, independently of the presence of tubercles in the lungs. On the contrary, it is well known that hæmoptysis may be the consequence of disease of the heart or large blood-vessels, and more especially the consequence of deranged menstruation, and other obvious morbid states; but when none of these causes are present, and when it occurs repeatedly, either before or after the supervention of cough, it is of all the symptoms at the commencement or early stage of phthisis, by far the most important as indicating the existence of the disease. Of the value of this symptom of phthisis you will consult with advantage the work of M. Louis, whose labours on the pathology of this disease will afford you much practical diagnostic information.

I may, however, further observe, in regard to this important symptom, that although it most frequently occurs in patients who have, as in the case of our patient, been subject for a variable length of time to cough, it is not unfrequently met with in others who have never had cough; who are, in fact, to all appearance, in the enjoyment of perfect health; and in such cases, and in the absence of other causes, is the first symptom of incipient phthisis, or of the presence of tubercles in the lungs; and I may further notice the fact that hæmoptysis is a very rare occurrence, indeed, in catarrh or bronchitis, a fact which gives additional value to its presence as a diagnostic symptom of phthisis.

I have said that the local signs furnished by percussion and auscultation in this case, greatly increased the diagnostic value of the hæmoptysis as a symptom of the early stage of tubercular phthisis. These local signs were limited to the right side of the chest, within a circumscribed space under the clavicle. They consisted of some dulness on percussion; a rough or harsh sound during inspiration; and of a sound nearly as strong, and much of the same character, during expiration. All of you are, no doubt, familiar with the value of the first sign, or dulness on percussion in the subclavicular region, as indicating the existence of tubercles in this region of the chest, this being so frequently the primary seat of these bodies as to constitute a law in regard to their relative frequency in the pulmonary organs. Here, however, the dulness was so slight that it might have been overlooked, or its importance might have been underrated, but for the harshness of the sound which accompanied inspiration, and more especially the existence of an analogous sound during expiration. You are aware that in the healthy state of respiration the sounds which accompany inspiration are the vesicular, bronchial, and tracheal; that those which accompany expiration are the bronchial and tracheal. There is no vesicular murmur heard, and even very little of the bronchial sound, during healthy expiration. The reason why the sounds alluded to are produced with greater intensity, and in greater number, in inspiration than in expiration, is sufficiently obvious if we reflect on the mechanism of respiration. During inspiration the air, as has been remarked by Dr. Williams, is the moving body, and entering the lungs with considerable velocity, impinges against the angles and sides of the bronchi and cells which it has to dilate, and must give rise to sound throughout the whole course of its passage. During expiration, the air, on the contrary, is put in motion by the compressed and contracting lungs, and yielding passively to this cause, does not acquire motion or resistance enough to produce sound, until, by the converging together of the small tubes, it is gathered into a current in the larger tubes, where, impinging against their sides with its now acquired velocity, it at length produces sound. Hence you can perceive why sound is heard only towards the middle or termination of the act of expiration, and heard only in bronchi of a certain size; and why it should possess a somewhat hollow and blowing character, rather than the diffuse, soft, vesicular murmur of inspiration, heard over the greater part of the surface of the chest.

I have mentioned these circumstances in regard to the production of sound during inspiration and expiration, that you might perceive more clearly the value of the sign observed in this patient manifested during the latter act. Instead of being feeble, the sound of expiration is increased, and nearly as loud as that of inspiration. Now, what is the cause of this, and how is it occasioned? That it depends on the presence of tubercles there seems to be now no longer the slightest doubt; Louis, Andral, and others, having verified the fact since it was first pointed out by the late Dr. Jackson, a young American student, while studying the stethoscopic signs of phthisis in Paris.

As to the manner in which this sound is produced, or becomes perceptible to the auscultator, the following is the explanation given of it by Dr. Jackson:—

"As soon as tuberculous matter is deposited there exists a solid material around the bronchi, which will transmit the sound made by the passage of the air through these tubes. But at this early period of the disease a certain portion of the lung in the part affected is still permeable to the air, and, therefore, the murmur of vesicular expansion, during inspiration, entirely masks the sound of the air passing through the bronchi, which would otherwise have been transmitted through the denser surrounding medium. During expiration, however, circumstances have changed; the air, on passing through the bronchi, produces the same sound as on its entrance; and, as now there is no vesicular expansion to mask it, it is easily transmitted through the diseased or condensed part to the ear of the observer."

How far this explanation of the sound in question be the correct one, is a matter of no great importance; its occurrence, under the circumstances which I have mentioned, at an early stage of phthisis pulmonalis, and as indicating the presence of tubercles in the portion of the lung in which it is heard, is certainly one of the most important of the physical signs of this disease.

Before leaving this case, I may direct your attention to the sputa, which are peculiarly characteristic of the early stage of phthisis, when not complicated with bronchitis or pneumonia. In this patient they form a striking contrast to those observed in the other patient, in whom the disease is much farther advanced. They consist of a gray or pearly-coloured substance, of the consistence of tough mucus or boiled albumen, semi-transparent, collected into rather small irregular masses, swimming in a moderate quantity of a clear, watery-looking fluid, or slightly adherent to the vessel in which they are contained. The quantity of the sputa may vary considerably, but in general is not great, and in our patient is small, and is coughed up with considerable difficulty.

These, then, are the principal circumstances to which I have been desirous of directing your attention in this case, viz., the characters of the sputa, the physical signs detected by percussion and auscultation, as signs of the early or incipient stage of pulmonary phthisis, and the hæmoptysis by which it is so frequently preceded.

I shall now make a few remarks on the principle of the treatment which I have adopted in this case; and I may first observe that the case is one which, as regards the stage of the disease, and its limited extent, offers a fair chance of success to any plan of treatment which has received the approbation and recommendation of practical physicians. The plan of treatment to which I now shall allude is that which consists chiefly in the frequent use of *emetics*. Whatever may have been the theory of the disease which suggested this plan of treatment, it was, at a remote period, adopted and recommended by several eminent physicians, as the most successful; and numerous cures of phthisis are reported as having been accomplished under its judicious management. It is a method, however, which was never generally adopted, and must, no doubt, have often sadly disappointed both the patient and the practitioner; and, besides, when we reflect on the very imperfect means which the physician, at the time this practice was most in vogue, possessed of determining the existence of phthisis pulmonalis, or of discriminating between this so frequently fatal disease, and other usually curable diseases of the chest, with which he must frequently have confounded it, we cannot place much reliance on the curative effects of the emetic plan of treatment under such circumstances. No modern physician, qualified for the task, that is to say, capable of establishing the diagnosis of tubercular phthisis by means of its *physical signs*, has, as yet, so far as I am aware, given this plan of treatment a fair trial, or furnished us with facts deserving of the slightest confidence.

Only seven years ago, a modern physician, certainly, published a small treatise, entitled "Observations and Experiments on Phthisis Pulmonalis, followed by a particular Method of Cure of this Disease." The author of this treatise is a Neapolitan, Giovanni de Vitis, first physician of the Military Hospital; and when I first heard of the number of cases of cure of phthisis which he states to have accomplished, my feeling of disbelief was so strongly excited that I gave but a very limited credence to the statement, and this I was the more disposed to do, that I had learned from personal observation the incapacity of physicians in the Neapolitan and Roman States of discriminating between phthisis and some other curable affections of the chest, more especially chronic catarrh and its complications, and chronic pleurisy. It is only within these few days that I have been able to procure the work of this author, and the perusal of it has more than confirmed the statement I have just made as to the utter worthlessness of his authority on such a subject, and the results which he has published of his new method of treatment. He does not even seem to know that such means as percussion and auscultation are employed to detect the existence of the disease; and yet, according to the old symptomatological system, he boldly classes the cases he has submitted to his method of treatment, under the heads—First, of chronic catarrh; second, of phthisis in the first degree; third, of phthisis in the second degree; fourth, of phthisis in the third degree. Of these he says he has cured forty cases of chronic catarrh; forty-seven of phthisis in the first degree; one hundred and two of phthisis in the second degree; and twenty-seven of phthisis in the third degree.

I shall not detain you by detailing the symptoms of any of the cases he has published, or the length of time which the treatment lasted, and for the reason I have already stated, that no confidence can be placed in statements regarding the cure of a disease such as phthisis, unless we possess the most satisfactory evidence in the first place of the existence of the disease before the treatment, and of its non-existence at some subsequent period. However, it is on other grounds than those I have already alluded to, that I am disposed to attach some importance to the treatment of phthisis by emetics. If the result of my researches on the seat and nature of tubercle is founded on fact, it affords some grounds for the rational hope that the cure of pulmonary phthisis may be promoted or facilitated by the employment of emetics. If, as I have endeavoured to prove, the tuberculous matter which constitutes the material cause of the disease, is contained principally, or in the great majority of cases, within the air cells and minute bronchi, it is easy to perceive that its expulsion will be effected or promoted by the employment of such means; that the destruction of the pulmonary tissue will be less likely to occur, or occur less extensively; and that time may be afforded for the correction or the removal of that state of the constitution on which the formation of the tuberculous matter essentially depends, and without which the cure of the disease would in vain be attempted. The employment of emetics, under circumstances so unfavourable as those in which patients are placed in hospitals, will, I am afraid, be attended by disappointment, as, whatever efficacy they may possess in effecting the dislodgment and expulsion of the tuberculous matter, we cannot, at the same time, obtain that assistance from other means derivable from the well-regulated influence of temperature or climate, including pure air, exercise, and a variety of hygienic conditions, which conjointly contribute to the same end, and more especially towards the removal of the tuberculous diathesis.

As, however, the case which has given occasion to these remarks present a favourable opportunity for trying the efficacy of emetics, half a grain of the tartarized antimony, in solution, has been ordered to be taken every morning, and to be repeated, if necessary, until vomiting has been produced. The bowels to be regulated by the occasional use of a calomel and a colocynth pill; and for the present the patient is to be confined to low diet. The result of the treatment will be made known to you, and I wculd request you to observe for yourselves, and to examine the chest of the patient with care, that you may fully appreciate the importance of the physical signs which announce in him the existence of phthisis.

Having dwelt so long on this subject, I shall be brief with the observations I have to offer you on the second. I have already stated that this patient, James Shirly, æt. 36, of a stout, muscular form, never had a day's illness before the present attack, about two months and a half before his admission into the hospital, the 30th of this month; and yet how different the state of this patient from that of William Calvert, whose case I have laid before you, in whom the commencement of the disease dates only about two weeks earlier. In this patient it has, as I have stated, arrived at the second stage; the extent of the tubercular deposit is much greater, exists on both sides of the chest; but on the right side, the same side as in the other case, the signs of softening of the tuberculous matter, aud of tubercular excavations, were recognisable more than two weeks ago, at the time he was admitted into the hospital.

The history of this case is very similar to that of the other. The patient caught cold, and soon became subject to a slight cough, which gradually increased; and, in the early part of Christmas, was followed, for the first time, and after a fit of severe coughing, by the discharge of about a tea-spoonful of blood. A week after, he spit about the same quantity of blood after coughing. On the 2d of January after a paroxysm of coughing, he brought up nearly a pint of blood, and, two days after, to the amount of two pints. After this period he spat up, on two separate occasions, small quantities only of blood. Having gone for advice to a dispensary, he had a few leeches given him to be applied to the chest, and some days after, was cupped on the same part, apparently without any relief to the cough, which is said to have gradually increased, whilst the expectoration, at the same time, became more profuse.

On admission, the cough was very troublesome; worse at night, as is generally the case; it was accompanied by the expectoration of a quantity of thick, greenish-yellow coloured, rather tenacious mucus, somewhat frothy, and sometimes tinged with blood, and brought up with difficulty. On examination, sonorous, sibilant, and mucous rattles were heard in various parts of the chest. Besides, there was heard a slight gurgling sound under the right clavicle, dulness on percussion on the posterior part of the chest, opposite and over the clavicle, and occasionally a cavernous ring on coughing. The sounds of the heart were normal; pulse 110; skin hot and dry during the day, but perspiring during the night, and sometimes profusely; tongue whitish, and slightly furred; throat sore, from constant coughing; appetite good; no sickness; bowels open from medicine; urine less than in health, and deposits a sediment.

I shall merely observe that in these symptoms you have, first, the signs of bronchitis, and, secondly, those of phthisis. The yellowish-green coloured expectoration; mucous, sonorous, and sibilant rattles, indicated the presence of the bronchitis, and that of a subacute character, and which appears to have existed for some time. The dulness on percussion, the gurgling sound, and cavernous ring, are, in this case, equally characteristic of the presence of tubercular phthisis, not merely because of the existence of these signs, but because of their being heard at the summit of the upper lobe of the lung, and for the reasons with which you are already acquainted.

This patient had already lost much in strength and flesh; and that roughness of the voice, so common in confirmed phthisis, was also very conspicuous, and has of late become more so. He has been three weeks in the hospital, and has obviously become weaker and leaner; the cough remains much the same; expectoration is somewhat more easy; the sputa, although perhaps not so great in quantity, has more of the puriform character, is sometimes thick and adhesive, indicating the persistence of the subacute bronchitis, and are still tinged with blood; and the night perspirations are more profuse.

On the admission of the patient, cupping between the shoulders was employed, and was followed by some relief to the cough; and, during the first week, he took an expectorant and anodyne mixture during the day, and an opiate at night. During the second week he was put upon the use of the solution of tartarised antimony, to be taken every morning. This was followed each time by vomiting, and the expectoration of a considerable quantity of muco-puriform matter. As, however, no amendment appeared to take place under the use of this emetic solution, and as the patient complained of great fatigue from its operation, and also of weakness, it was laid aside.

I cannot conclude without remarking that there is little hope of being able to do any permanent good in this case. If the disease has not advanced since the admission of the patient, it has exhibited no symptom, as I have already said, of amendment. The extent of the hæmoptysis in the first period of the disease is generally an index of the extent of the tubercular deposition, and as it was very considerable in this case, the more unfavourable is the prognosis, independently of the other circumstances which I have named as leading to the same conclusion.—*Lancet.*

---

*Two Cases of Episioraphy, to elucidate a Modification of the Operation.* By Dr. Fricke, of Hamburg. *Case* 1.—This woman had already been successfully operated upon about fifteen months before, on account of a considerable prolapsus of the uterus. About two months before her admission, on the 8th March, 1837, she had been delivered of a full-grown child; but the accoucheur who had been called in to assist her had judged it necessary to divide the adhesions, consequent on the former operation, in order to facilitate labour. The natural result was a second prolapsus to an extent which prevented all exertion.

The edges of the labia were made raw and approximated to each other, and held in position by a number of sutures. The modification in the operation consisted in leaving a free space (in this case accidentally produced) of about half an inch in extent, at the anal commissure, by which the menstrual and other secretions could escape, without accumulating and causing inconvenience. Six weeks after the operation the woman left the hospital completely relieved.

*Case* 2.—J. A., aged forty-one, admitted 6th July, 1837, had suffered during the last six years from prolapsus of the uterus, which followed on difficult parturition, during which the forceps were applied. The protruded uterus and vagina were about the size of a child's head; the mucous membrane of the vagina was smooth, dry, and leathery, excoriated in many parts, and covered with hard scabs. Several days' rest, and preliminary treatment, were necessary before the uterus could be replaced; and seven days afterwards, the menses, which had not appeared for years, began to flow.

On the 22d August, the operation was performed as described in the preceding case, except, that here a space, sufficient to admit the finger, was purposely left free at the anal commissure. The union was complete in fourteen days, except a small part at the posterior edge of the wound, which speedily healed on touching it occasionally with caustic. The prolapsus was completely retained.

The advantages resulting from this modification of the operation, are:

1. It is less painful, and is more quickly performed.
2. Local antiphlogistic remedies are more easily applied.
3. Injections into the vagina escape readily through the orifice.
4. Mucus and the menstrual fluid are not retained.—*Brit. and For. Med. Rev., from Zeitschrift für die gesammte Medicin.*

---

*Influence of Artificial Feeding upon the Mortality of Infants.*—It would appear that the establishment of beneficent institutions in France, for the reception of illegitimate and deserted children, has not only tended to encourage profligacy, licentiousness, and cruelty, but has at length "fructified," after its own kind, in the production of still greater enormities. Such an evil tree could not but bring forth evil fruit.

It was soon found in the institutions alluded to, that the compassionate regulations of the "tours d'arrondissement," by which the wretched infants were sometimes enabled to obtain their natural food, and wholesome exposure to the air, so greatly increased the living burdens of the charity, that this mercy was withdrawn. And now the public are coolly informed of the statistical difference between natural and artificial feeding upon the mortality of infants; as if this artificial feeding had no cause in the moral wrong done to the infants by the prohibition of the only salutary course which had hitherto been open to them.

M. l'Abbé Guillard, chaplain to the General Hospital of Tours, states, in the hospital of X, (which he deems it inconvenient to name,) not a single infant has been suckled; all those who are received are nourished by hand, through a sucking bottle. In this hospital, a very exact account has shown that last year, out of 244 children, 197 had died at the end of the year! Of these, 116 lived only from a day to a month. Out of 127 infants in 1834, there remained only 29 at the end of the year. On the 1st of January, 1835, out of 362 infants, there remained alive only 127 at the expiration of twelve months.

The suppression of the "tours" has shown, also, in other hospitals, the murderous effect of this prohibition. Thus, in the Hospital de Poitiers, where the habitual proportion of deaths, in the first month of life, had been 12 to 100, have been now increased in that month above 35 in 100. In the Hospice de Loudun, 2 only out of 11 remained alive at the end of the year, and 4 out of 9 received in the first six months of 1835. These had been all artificially fed, and prohibited from being taken out.

At Moulins, in the first months of 1835, 128 were admitted, and 100 of these had died.—*Ibid, from Bulletin Général de Thérapeutique.*

